# Investigation of Volatile Compounds, Microbial Succession, and Their Relation During Spontaneous Fermentation of Petit Manseng

**DOI:** 10.3389/fmicb.2021.717387

**Published:** 2021-08-12

**Authors:** Yanqin Ma, Tian Li, Xiaoyu Xu, Yanyu Ji, Xia Jiang, Xuewei Shi, Bin Wang

**Affiliations:** Food College, Shihezi University, Shihezi, China

**Keywords:** Petit Manseng, spontaneous fermentation, fungal community, volatile composition, correlation analysis

## Abstract

Petit Manseng is widely used for fermenting sweet wine and is popular among younger consumers because of its sweet taste and attractive flavor. To understand the mechanisms underlying spontaneous fermentation of Petit Manseng sweet wine in Xinjiang, the dynamic changes in the microbial population and volatile compounds were investigated through high-throughput sequencing (HTS) and headspace solid-phase microextraction (HS-SPME) coupled to gas chromatography-mass spectrometry (GC-MS) technology, respectively. Moreover, the relationship between the microbial population and volatile compounds was deduced *via* multivariate data analysis. *Candida* and *Mortierella* were dominant genera in Petit Manseng wine during spontaneous fermentation. Many fermentative aroma compounds, including ethyl octanoate, isoamyl acetate, ethyl butyrate, ethyl decanoate, isoamyl alcohol, ethyl laurate, isopropyl acetate, hexanoic acid, and octanoic acid, were noted and found to be responsible for the strong fruity and fatty aroma of Petit Manseng sweet wine. Multivariate data analysis indicated that the predominant microorganisms contributed to the formation of these fermentative aroma compounds. *Hannaella* and *Neomicrosphaeropsis* displayed a significantly positive correlation with the 6-methylhept-5-en-2-one produced. The current results provide a reference for producing Petit Manseng sweet wine with desirable characteristics.

## Introduction

Wine is an alcoholic beverage derived from grapes, and its production involves chemistry and biology (Belda et al., 2017). Based on its sugar content, wine can be classified into dry, semi-dry, sweet, and semi-sweet wine (Brainina et al., 2004). Because of their taste and rich flavor, sweet wines are popular (Swami et al., 2014). The grape varieties used to make sweet wine mainly include Riesling, Sauvignon Blanc, and Petit Manseng (Englezos et al., 2018; Lan et al., 2021). Of these, Petit Manseng is a thick-skinned, white grape variety grown in southwest France, and it is widely used to produce high-quality sweet wines rich in a fruity, slightly spicy aroma (Gardner et al., 2017). As a late-harvest cultivar, Petit Manseng has a high sugar and acid content, and consequently, it is strongly resistant to several fungal diseases (Robinson et al., 2013).

A wine’s quality is determined based on its taste (Loureiro et al., 2016), color (Zhang et al., 2018), and aroma (Sherman et al., 2020). An important component of wine, the aroma is considered to be indicative of the regional and cultivar characteristics of a wine. This is because aroma is affected by natural elements (e.g., grape cultivars and climatic condition) (Mirás-Avalos et al., 2017), wine-making techniques (e.g., oak maturation and fermentation method) (Crump et al., 2015; Shi et al., 2019), and indigenous microorganism (e.g., bacteria, yeast, and fungi) (Liu et al., 2017). Wine aroma can be divided into varietal, fermentative, and aging (Belda et al., 2017). Varietal aromas, derived from wine grapes, are responsible for most of the aromas in a wine (Waterhouse et al., 2016). For example, when homologous, *Cabernet Franc*, *Cabernet Sauvignon*, *Sauvignon Blanc*, *Merlot*, and *Carmenère* have a typical bell pepper, vegetal, and earthy aroma (Ruiz et al., 2019).

High-throughput sequencing (HTS) is useful for evaluating dynamic changes in microbial populations during fermentation (Guo et al., 2020). Therefore, HTS has enabled the identification of a variety of dominant microorganisms, especially culture-independent microorganisms, in wine (Bučková et al., 2018). Wine is fermented by various indigenous microorganisms, and these vary based on the wine grape cultivar (Francesca et al., 2016). For instance, *Hanseniaspora*, *Metschnikowia*, *Saccharomyces*, *Lactococcus*, and *Leuconostoc* are predominant in Grüner Veltliner ice wine (Alessandria et al., 2013), whereas *Leuconostoc*, *Lactococcus*, and *Saccharomyces* are dominant in “Marselan” red wine (Lu et al., 2020). Among the aforementioned indigenous microorganisms, *Saccharomyces cerevisiae* not only promotes alcoholic fermentation but also increases phenol and antioxidant content through alcoholic fermentation (Caridi et al., 2017). Non-*Saccharomyces* yeasts are gaining increasing attention because they can improve the aroma complexity or mouth-feel of wines (Padilla et al., 2016).

Based on whether or not a starter culture is used, wine fermentation can be divided into inoculated fermentation and spontaneous fermentation. Compared with inoculated fermentation, spontaneous fermentation, which is caused by complex indigenous microorganisms, can provide a more complex and richer flavor in wine (Lu et al., 2021). Spontaneous fermentation makes full use of diverse microorganisms, especially the specific autochthonous yeasts (de Celis et al., 2019). The spontaneous fermentation by non-*Saccharomyces* yeasts has been found to enhance the aroma character of Cabernet Sauvignon, Chardonnay, and Vidal wines (Lu et al., 2020).

High-throughput sequencing (HTS) combined with multivariate data analysis has been widely used to investigate microbial diversity and its impact on wine quality (Böhmer et al., 2020). An increasing number of microorganisms, contributing to potential wine flavors, including *Hanseniaspora uvarum*, *Wickerhamomyces anomalus*, and *Lachancea thermotolerans*, have been identified (Belda et al., 2016).

Petit Manseng sweet wines are popular among young consumers (Chaplin, 2011). Although the sensory characteristics of Petit Manseng wines have been investigated *via* headspace solid-phase microextraction (HS-SPME) coupled to gas chromatography-mass spectrometry (GC-MS) and by using a polymer electronic nose (Gardner et al., 2017), the relationship of volatile compounds with the microbial community in these wines during spontaneous fermentation remains unclear. To understand the mechanisms underlying the spontaneous fermentation of Petit Manseng sweet wine in Xinjiang, dynamic changes in the microbial community and volatile compounds during spontaneous fermentation were investigated through HTS and HS-SPME/GC-MS, respectively. The relationship between the microbial populations and the volatile compounds was elucidated using multivariate data analysis. The current results provide a theoretical reference for producing Petit Manseng sweet wines with typical characteristics.

## Materials and Methods

### Wine Making and Sampling

Petit Manseng was harvested from Changji County, Xinjiang Province, China in October 2019. After strict screening, healthy grapes were immediately crushed. The biochemistry of these grapes was then analyzed: residual sugar content, 347.35 g/L; yeast assimilable nitrogen (YAN): 347 mg N/L; pH, 3.49; soluble solids, 32.6 °Brix; total acidity, 5.17 g/L. This was followed by the addition of 20 mg/L pectinase. The grapes were macerated at 8−10°C for 48 h; then, the separated grape supernatants were subjected to alcoholic fermentation. All fermentation was performed in 50-L fermenters at 15–17°C (factory ambient temperature) under static conditions for 14°days. The alcohol fermentation was considered to be completed when residual sugar content was <110 g/L. The fermentation liquid (250 mL) was collected on fermentation days 0, 1, 4, 7, 11, and 14. All samples were centrifuged at 1,000 × g at 4°C for 10 min. The precipitates were then collected and used for HTS analysis, whereas the supernatants were used to assess volatile compound analysis. All samples were stored at −80°C until analysis. Each sample was analyzed in triplicate.

### Determination of Physicochemical Properties

During alcoholic fermentation, some fermentation parameters were measured including pH, total acidity, and residual sugar and ethanol content. The pH of wine was measured using a calibrated pH meter (PHS-3C; Shanghai Jingke, Shanghai, China). Residual sugar content was assessed using dinitrosalicylic acid (DNS) method (Miller, 1959). Total acidity and ethanol content were determined according to the national standard of China: GB/T 15038-2006, “Analytical methods of wine and fruit wine.” Organic acids in the wine samples were analyzed through high-performance liquid chromatography (HPLC) using previously described methods with some modifications (Murtaza et al., 2017). Each sample was centrifuged and filtered through a 0.22-μm filter, and the organic acids were identified through HPLC equipped with a Spursil C18 column (250 mm × 4.6 mm × 5 μm; Dima Technology, Guangzhou, China). A mixture of 0.1% phosphoric acid-methanol was regarded as the mobile phase, with a flow rate of 1 mL/min. The UV detection wavelength was maintained at 210 nm, and the column temperature was 40°C. Each sample was measured in three replicates.

### Fungal Isolation and Identification

The wine sample was serially diluted from 1 × 10 to 1 × 10^5^ times by using sterile water and plated on yeast extract-peptone-dextrose (YPD) agar, and all plates were cultured at 28°C for 3 days under sterile conditions. After incubation, the fungal colonies on each plate were selected and isolated as representative fungal populations present during spontaneous fermentation. All representative strains were incubated on the WL nutrient agar (Haibo, Qingdao, China) at 28°C for 5 days. Then, fungal colonies with different morphological types were selected for the next step of identification. Single colonies of fungi were purified and stored in YPD liquid medium with 40% glycerol at −20°C. Fungal strains were analyzed and identified by comparing nucleotide sequences in the GenBank database (NCBI: https://blast.ncbi.nlm.nih.gov/blast.cgi).

### HTS

The total DNA of fungal colonies isolated and selected from our wine samples was extracted using the CTAB/SDS method (Lin et al., 1994; Brandfass and Karlovsky, 2008). Next, the DNA of microorganisms were sent to Novegene Company (China) for PCR amplification and HTS. The primers used were ITS5-1737F (5′-GGAAGTAAAAGTCGTAACAAGG-3′) and ITS2-2043R (5′-GCTGCGTTCTTCATCGATGC-3′). For PCR, the total reaction volume was 25 μL, comprising 15 μL of the PCR Master Mix, 2 μL of the forward and reverse primers, 10 ng of the template DNA, and water (PCR-grade) to adjust the volume. We used the following thermal cycling program: initial denaturation at 98°C for 1 min, followed by 35 cycles of denaturation at 98°C for 10 s, annealing at 50°C for 30 s, and elongation at 72°C for 30 s, and finally, extension at 72°C for 5 min. All PCR products were detected through 2% agarose gel electrophoresis and purified using the Qiagen Gel Extraction Kit (Qiagen, Germany). Next, sequencing libraries were generated using TruSeq DNA PCR-Free Sample Preparation Kit (Illumina, United States). The library was assessed on a Qubit 2.0 Fluorometer (Thermo Fisher Scientific) and Agilent Bioanalyzer 2100. The Illumina NovaSeq platform was used to sequence 250-bp paired-end reads.

### Processing of Sequence Analysis

Paired-end reads were assigned to samples based on their unique barcodes and truncated by cutting off the barcodes and primer sequences. Paired-end reads were merged using FLASH (version 1.2.7). The raw reads were filtered to obtain high-quality clean reads using QIIME (version 1.9.1). Moreover, the chimeric sequences were filtered out by comparing them with those in the SILVA database (a reference database). Moreover, the same operational taxonomic units (OTUs) were clustered from sequences with 97% sequence similarity by using Uparse (version 7.0.1001). The most abundant sequence in each OTU was selected as the representative sequence. Taxonomic units were assigned to the representative sequence (i.e., to each OTU) using the SILVA database. MUSCLE (version 3.8.31) was used for multiple sequence alignment and studied the phylogenetic relationship of different OTUs. Finally, alpha diversity indexes such as Shannon, Simpson, Chao1, and ACE were calculated on QIIME (version 1.9.1).

### Determination of Volatile Compounds

Headspace solid-phase microextraction (HS-SPME)/gas chromatography-mass spectrometry (GC-MS) was used to extract and analyze volatile compounds in our wine, by using Kong’s et al. (2019) method with some modifications. In brief, 1 g of NaCl was added to 5 mL of sample supernatants. This mixture was then transferred to a 20-mL HS vial, followed by 1 μL of 3-octanol (165 mg/mL) as the internal standard. The vial was sealed immediately with a polytetrafluoroethene (PTFE) silicone diaphragm and balanced at 40°C for 10 min. An SPME fiber (DVB/CAR/PDMS 50/30 μm; Supelco, Bellefonte, PA, United States) was then inserted into the sealed glass vial and exposed to the HS for resolution for 40 min at 40°C. After extraction, the fiber was inserted into the GC injection port at an interface temperature of 250°C for 5 min. Each volatile compound was analyzed using an HP INNOWAX column (30 m × 0.25 mm; Agilent). Helium was the carrier gas, circulated at 1 mL/min, and the electron ionization energy was maintained at 70 eV. The scanning range of the total ion chromatographs was 35–350 m/z. The temperature program was as follows: 3 min at 50°C, followed by 2°C/min to 100°C, 4°C/min to 180°C, and 10°C/min to 230°C.

The volatile aroma compounds were identified by matching their retention indexes with those in the mass spectra library (NIST). Moreover, the retention indexes were evaluated using the Kovats system (Zenkevich, 2002). All compounds were semiquantified as 3-octanol equivalents. The semi-quantitative data of the aroma compounds were calculated as follows:

(1) $$\text{RCAC}\,\left(\mu \,g/L\right)=\frac{\text{PAVC}}{\text{PAIS}}\times \mathrm{CIS}\,\left(\mu \,g/L\right)$$

where RCAC is the relative concentration of the volatile compounds, PAVC is the peak area of volatile compounds, PAIS is the peak area of internal standard, and CIS is the final concentration of internal standard.

### Sensory Analysis of Wine

In this study, the Petit Manseng grapes were used as raw materials for vinification, we also compared them with the Petit Manseng wine. Sensory analysis was performed using Belda’s et al. (2015) method with some modifications. In brief, the wine was assessed by 10 trained panelists (6 female and 4 male) from Shihezi University. During the formal sensory analysis process, the team members were asked to form consistent terminology by consensus and described the sensory notes. Sensory descriptions included fruity, floral, green, acidity, sweetness, fatty, and bitterness, all scored from 0 (weak) to 10 (intense).

### Statistical Analysis

For each sample group, three parallel samples were analyzed. The differences between our samples were analyzed using analysis of variance with Duncan multiple tests, with the significance level set at 5% (*P* < 0.05) on SPSS (version 20; IBM, Chicago, United States). R (version 4.0.4) was used to produce a heat-map for the microbial populations and volatile compounds. The correlation among microorganisms and volatile compounds was determined multivariate data analysis, including bidirectional partial least squares (O2PLS), on the software program SIMCA (version 14.1). The linear correlation coefficient between the selected microbial genera and volatile compounds was visualized *via* O2PLS on Cyto-scape (version 3.6.1).

## Results

### Physicochemical Properties During Spontaneous Fermentation

**FIGURE 1:**
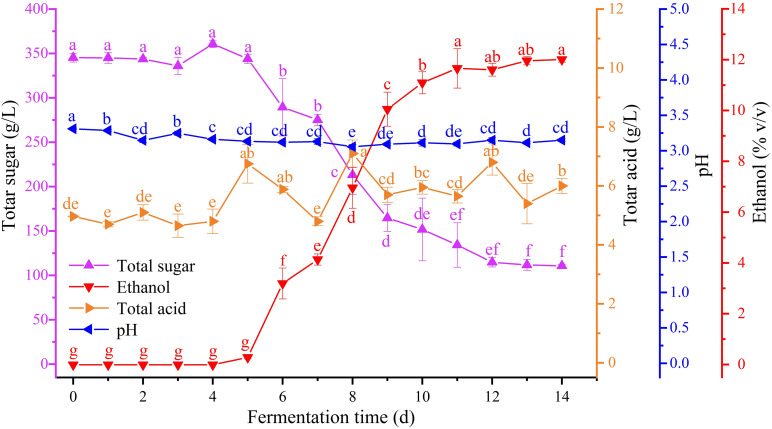
Change of physicochemical indexes in different fermentation time: total sugar (purple), ethanol (red), total acid (orange), and pH (blue) (for interpretation of the references to color in this figure legend, the reader is referred to the Web version of this article).

Dynamic changes in the ethanol, total sugar, and total acid content and the pH of zymotic fluid from six periods during Petit Manseng spontaneous fermentation were detected (Figure 1). The total sugar content of Petit Manseng juice reached up to 345.35 g/L. During Petit Manseng spontaneous fermentation, sugar in the zymotic fluid was converted to ethanol. Thus, total sugar content sharply decreased after 4 days of fermentation and remained at 110.84 ± 1.69 g/L on the 14th day. Then, ethanol concentrations increased after 4 days of fermentation and reached up to 12.01 ± 0.09% (v/v) on the 14th day. Total acid content fluctuated from 4.97 ± 0.02 to 6.00 ± 0.26 g/L. The pH remained relatively stable at 3.2 throughout the fermentation process.

The organic acid content directly affects the flavor and taste of white wine (Nie et al., 2017). In this study, the organic acids detected from Petit Manseng were tartaric acid, lactic acid, acetic acid, citric acid, and succinic acid (Table 1). Tartaric acid content sharply decreased throughout the fermentation process, with it almost depleted at the end of fermentation. In contrast, lactic acid content showed a slight increase, with a final value of 0.04 ± 0.00 mg/mL. Acetic acid content showed an upward trend overall, increasing sharply on the 11th day and then slowly decreasing to finally reach a concentration of 0.09 ± 0.01 mg/mL. Citric acid showed an upward trend overall. In particular, citric acid was continuously consumed and synthesized during spontaneous fermentation. Finally, succinic acid content remained relatively stable throughout the fermentation process.

**TABLE 1:** Organic acid content in the wine (mg/mL).

Organic acids	A	B	C	D	E	F
Tartaric acid	0.36 ± 0.02^a^	0.28 ± 0.01^b^	0.31 ± 0.02^ab^	0.20 ± 0.03^b^	0.19 ± 0.04^b^	0.03 ± 0.00^c^
Lactic acid	0.03 ± 0.00^b^	0.02 ± 0.00^b^	0.00 ± 0.00^c^	0.00 ± 0.00^c^	0.04 ± 0.00^a^	0.04 ± 0.00^a^
Acetic acid	0.04 ± 0.00^c^	0.05 ± 0.00^c^	0.05 ± 0.01^c^	0.06 ± 0.01^b^	0.10 ± 0.00^a^	0.09 ± 0.01^a^
Citric acid	1.07 ± 0.03^b^	1.13 ± 0.05^b^	1.09 ± 0.03^b^	1.13 ± 0.03^b^	1.26 ± 0.02^a^	1.22 ± 0.00^a^
Succinic acid	0.28 ± 0.01^a^	0.30 ± 0.02^a^	0.28 ± 0.00^a^	0.24 ± 0.01^b^	0.23 ± 0.01^b^	0.22 ± 0.00^b^

*Data are expressed as the means ± standard (*n* = 3). The different lowercase letters in each row indicate a significant difference between the samples (*P* < 0.05).*

### Volatile Compounds During Spontaneous Fermentation

Volatile compounds from six fermentation periods of Petit Manseng wine during spontaneous fermentation included 17 esters, 19 alcohols, 9 acids, 4 aldehydes, 4 ketones, and 3 other volatile compounds (Table 2).

**TABLE 2:** Dynamic changes of flavor compound content during Petit Manseng fermentation process (μg/L).

Compounds	RI	A	B	C	D	E	F	Odor threshold (μg/L)	OAV^2^	Odors^1^
**Esters**										
Ethyl acetate	888	4.91 ± 0.19^d^	3.83 ± 0.35^d^	104.27 ± 0.09^d^	842.00 ± 102.42^c^	1637.48 ± 285.66^b^	3170.56 ± 350.13^a^	7500 ^[A]^	0.1–1	Fruity, sweet ^[A]^
Isobutyl acetate	1000	ND	ND	3.49 ± 0.52^c^	16.85 ± 1.90^b^	32.89 ± 3.27^a^	35.92 ± 2.19^a^	1600 ^[B]^	<0.1	Strawberry, fruity, flowery^[A]^
Ethyl butyrate	1036	0.59 ± 0.11^c^	ND	3.53 ± 0.52^c^	122.38 ± 15.27^b^	313.73 ± 14.04^a^	293.68 ± 52.54^a^	20^[B]^	>1	Sour fruity, fruity, strawberry ^[B]^
Isoamyl acetate	1147	1.13 ± 0.16^d^	2.21 ± 0.94^d^	327.43 ± 32.69^d^	3893.87 ± 466.31^c^	5950.84 ± 390.73^b^	9052.43 ± 325.06^a^	30 ^[B]^	>1	Banana, sweet ^[B]^
Ethyl hexanoate	1233	1.78 ± 0.07^c^	4.28 ± 0.32^c^	1457.11 ± 14.16^b^	1557.17 ± 155.66^b^	3947.08 ± 416.23^a^	1283.17 ± 98.92^b^	5^[B]^	>1	Fruity, green apple, floral, violet^[B]^
Hexyl acetate	1272	0.31 ± 0.23^e^	17.10 ± 4.00^e^	671.51 ± 40.18^d^	1498.46 ± 257.60^b^	2463.10 ± 155.47^a^	1117.32 ± 29.89^c^	670^[B]^	>1	fruity, pear, cherry^[B]^
Propyl hexanoate	1293	ND	ND	4.52 ± 0.46^c^	7.48 ± 0.20^a^	4.12 ± 0.03^c^	5.64 ± 1.07^b^	nf		nf
3-Hexen-1-yl salicylate	1315	0.32 ± 0.01^e^	0.53 ± 0.12^e^	11.88 ± 1.10^d^	25.55 ± 2.38^c^	66.25 ± 0.42^a^	42.95 ± 2.20^b^	320 ^[Q]^	<0.1	nf
Ethyl octanoate	1435	0.41 ± 0.19^d^	7.13 ± 0.33^d^	929.25 ± 6.74^c^	5187.56 ± 309.43^a^	1825.99 ± 109.78^b^	1931.56 ± 25.46^b^	2 ^[B]^	>1	Fruity, pineapple, pear, floral^[B]^
Isopropyl acetate	882	ND	1.22 ± 0.19^c^	3.65 ± 0.30^c^	10.93 ± 1.16^c^	410.79 ± 1.77^b^	830.95 ± 21.3^a^	500^[I]^	>1	Fruity, unpleasant^[I]^
Ethyl decanoate	1638	0.37 ± 0.05^c^	2.03 ± 0.27^c^	552.21 ± 35.61^c^	2488.33 ± 106.52^b^	10138.64 ± 103.75^a^	2723.78 ± 655.16^b^	200^[C]^	>1	Fruity, pleasant ^[A]^
3-Methylbutyl octanoate	1122	ND	ND	1.52 ± 0.50^d^	24.16 ± 0.20^c^	35.26 ± 2.75^b^	41.86 ± 0.21^a^	nf		nf
Ethyl 9-decenoate	1694	ND	ND	23.84 ± 2.35^a^	15.46 ± 0.47^b^	13.41 ± 1.16^b^	4.21 ± 0.73^c^	100^[A]^	<0.1	Green, fruity, fatty ^[A]^
2-Phenylethyl acetate	1807	ND	ND	96.95 ± 0.05^d^	1053.22 ± 15.55^a^	627.80 ± 29.68^c^	770.53 ± 37.87^b^	250^[D]^	>1	Floral, roses^[D]^
Ethyl nonanoate	1531	ND	ND	3.41 ± 0.40^c^	5.48 ± 0.14^b^	6.27 ± 0.25^a^	6.64 ± 0.04^a^	1300 ^[B]^	<0.1	Waxy, fruity ^[B]^
Ethyl palmitate	2262	ND	ND	0.41 ± 0.04^d^	18.30 ± 0.88^a^	1.44 ± 0.08^c^	4.20 ± 0.14^b^	1000^[E]^	<0.1	Wax, fatty ^[F]^
Ethyl laurate	1835	0.59 ± 0.00^e^	1.71 ± 0.39^e^	552.21 ± 35.61^d^	3194.99 ± 112.38^b^	6140.38 ± 151.79^a^	2220.85 ± 56.09^c^	500^[E]^	>1	Sweet, floral, fruity, cream^[A]^
**Alcohols**										
1-Butanol	1142	ND	2.36 ± 0.42^c^	4.57 ± 0.47^c^	26.03 ± 0.12^c^	141.22 ± 1.66^b^	261.12 ± 46.36^a^	150000 ^[D]^	<0.1	Sweet, medicinal ^[D]^
1-Penten-3-ol	1159	0.71 ± 0.04^d^	1.09 ± 0.03^c^	4.33 ± 0.18^a^	3.33 ± 0.27^b^	4.19 ± 0.04^a^	ND	400 ^[Q]^	<0.1	nf
2-Penten-1-ol	1318	2.81 ± 0.26^d^	1.47 ± 0.32^e^	3.69 ± 0.06^d^	52.90 ± 0.65^a^	20.01 ± 0.01^b^	5.57 ± 0.59^c^	720 ^[Q]^	<0.1	nf
Ethanol	932	88.30 ± 0.56^e^	438.94 ± 5.26^e^	7421.74 ± 349.66^d^	27824.56 ± 2171.11^c^	76334.58 ± 2463.02^a^	44631.88 ± 2884.43^b^	950 ^[N]^	>1	nf
Isoamyl alcohol	1209	ND	341.79 ± 41.82^d^	1328.36 ± 186.88^d^	7238.78 ± 101.75^c^	23298.19 ± 1181.43^b^	36852.34 ± 4323.93^a^	30000^[B]^	>1	Whiskey, malt, burnt ^[B]^
3-Hexen-1-ol	1382	26.49 ± 1.65^d^	33.51 ± 2.37^c^	24.43 ± 2.10^d^	36.44 ± 3.87^c^	55.94 ± 4.45^b^	68.33 ± 0.02^a^	400^[J]^	0.1–1	Green grass, herb ^[C]^
2-Hexen-1-ol	1405	201.88 ± 1.08^a^	12.60 ± 0.26^b^	5.47 ± 0.08^d^	ND	6.84 ± 0.21^c^	7.90 ± 0.46^c^	400^[D]^	<0.1	Fruity, unripe banana ^[D]^
1-Hexanol	1355	429.47 ± 13.31^c^	790.79 ± 55.36^b^	756.59 ± 47.07^b^	736.84 ± 1.36^b^	399.44 ± 13.04^c^	1160.55 ± 83.06^a^	8000 ^[C]^	0.1–1	Green, herb^[B]^
1-Octanol	1557	ND	ND	21.49 ± 0.05^a^	9.70 ± 0.59^c^	13.43 ± 0.51^b^	ND	900 ^[A]^	<0.1	Flesh orange, rose, sweet herb^[A]^
1-Octen-3-ol	1450	11.71 ± 0.78^b^	18.35 ± 0.82^a^	ND	ND	ND	ND	10 ^[K]^		Mushroom ^[P]^
1-Decanol	1752	ND	ND	ND	7.99 ± 0.21^b^	9.00 ± 0.27^a^	1.69 ± 0.08^c^	400^[B]^	<0.1	Orange flowery, special fatty^[A]^
Terpinen-4-ol	1602	1.78 ± 0.25^c^	2.74 ± 0.28^b^	7.67 ± 0.11^a^	7.38 ± 0.64^a^	ND	0.84 ± 0.28^d^	5000 ^[E]^	<0.1	Light aroma, wood, soil ^[Q]^
Citronellol	1765	0.42 ± 0.25^e^	2.74 ± 0.28^d^	9.80 ± 0.19^a^	2.56 ± 0.27^d^	5.69 ± 0.08^c^	7.06 ± 0.08^b^	100 ^[B]^	<0.1	Green lemon ^[B]^
Benzyl alcohol	1870	2.00 ± 0.16^c^	3.38 ± 0.28^b^	5.31 ± 0.13^a^	5.24 ± 0.08^a^	0.90 ± 0.04^d^	ND	200000 ^[A]^	<0.1	Almond,fatty ^[A]^
1-Nonanol	1660	1.21 ± 0.13^c^	3.30 ± 0.35^c^	26.59 ± 3.37^a^	15.72 ± 0.16^b^	16.53 ± 0.25^b^	ND	600^[B]^	<0.1	Green ^[B]^
Phenylethyl alcohol	1906	9.47 ± 0.25^d^	26.09 ± 0.95^d^	244.90 ± 6.55^c^	1321.92 ± 177.12^a^	659.52 ± 38.09^b^	752.06 ± 19.2^b^	10000 ^[G]^	<0.1	Rose, sweet^[G]^
Undec-2-en-1-ol	1899	0.70 ± 0.08^c^	1.82 ± 0.09^b^	2.29 ± 0.37^a^	0.76 ± 0.12^c^	ND	ND	nf		nf
1-Pentanol	1250	3.35 ± 0.37^e^	1.70 ± 0.01^e^	15.67 ± 2.09^d^	19.53 ± 0.14^c^	32.65 ± 2.7^b^	37.67 ± 1.25^a^	64000 ^[E]^	<0.1	nf
2-Nonanol	1499	ND	ND	9.75 ± 0.26^c^	21.28 ± 0.20^b^	41.88 ± 2.68^a^	ND	30 ^[O]^	>1	Citric^[P]^
**Acids**										
Hexanoic acid	1846	8.09 ± 0.04^d^	35.82 ± 0.68^d^	364.81 ± 15.39^c^	1833.66 ± 87.26^b^	1807.23 ± 10.85^b^	2126.05 ± 77.99^a^	420 ^[A]^	>1	Cheese, rancid^[A]^
2-Methylhexanoic acid	1960	0.39 ± 0.09^e^	1.09 ± 0.20^e^	4.17 ± 0.14^d^	7.65 ± 0.18^b^	11.17 ± 0.16^a^	6.25 ± 0.31^c^	nf		nf
2-Hexenoic acid	1967	1.34 ± 0.45^e^	2.44 ± 0.04^d^	8.33 ± 0.06^a^	5.35 ± 0.06^b^	3.15 ± 0.19^c^	0.90 ± 0.15^e^	3000 ^[Q]^	0.1–1	Fatty^[Q]^
Octanoic acid	2060	5.77 ± 0.29^c^	62.87 ± 2.98^c^	1766.29 ± 28.36^b^	5567.66 ± 448.39^a^	1570.26 ± 277.62^b^	1322.85 ± 270.91^b^	500^[A]^	>1	Rancid, cheese, fatty acid ^[A]^
Dodecanoic acid	2498	ND	1.10 ± 0.14^e^	94.57 ± 3.30^b^	256.93 ± 8.72^a^	46.78 ± 4.03^c^	34.92 ± 0.11^d^	1000^[B]^	<0.1	Daurel oil flavor ^[B]^
n-Decanoic acid	2276	0.48 ± 0.13^d^	11.85 ± 1.21^d^	733.35 ± 9.95^b^	2135.69 ± 175.09^a^	498.38 ± 59.90^c^	684.66±110.50cb	1000^[M]^	0.1–1	Fatty, unpleasant ^[A]^
3-Methylbutanoic acid	1655	ND	3.48 ± 0.23^d^	8.82 ± 0.30^d^	21.13 ± 0.92^c^	38.14 ± 2.62^a^	30.30 ± 0.21^b^	33 ^[H]^	0.1–1	Rancid, cheese ^[H]^
Butanoic acid	1950	2.67 ± 0.25^d^	5.95 ± 0.43^c^	6.98 ± 1.19^c^	26.29±2.83ba	26.64 ± 1.58^a^	22.75 ± 1.32^b^	2200^[I]^	0.1–1	Sharp, cheesy, rancid ^[E]^
Nonanoic acid	2171	ND	1.07±0.21cb	1.50 ± 0.67^b^	5.56 ± 0.20^a^	4.63 ± 0.52^a^	4.96 ± 0.87^a^	500-800 ^[B]^	<0.1	Cheese, waxy flavor ^[B]^
**Aldehydes**										
Hexanal	1083	64.04 ± 2.5^a^	47.06 ± 0.65^b^	1.57±0.52dc	ND	3.08 ± 0.06^c^	ND	5-15^[B]^	0.1–1	Apple, green grassy ^[B]^
Hexen-2-al	1213	5.82 ± 0.11^d^	23.96 ± 0.90^b^	ND	40.23 ± 4.77^a^	14.78 ± 1.78^c^	8.55 ± 0.46^d^	24.2 ^[Q]^	0.1–1	Green, fruity ^[Q]^
Dodecanal	1711	0.81 ± 0.10^d^	1.31 ± 0.31^d^	2.05±0.21dc	3.26 ± 0.40^b^	8.50 ± 0.52^a^	2.84±0.27cb	0.13-0.29 ^[N]^	>1	Orange ^[N]^
Benzaldehyde	1520	1.05 ± 0.04^d^	4.38 ± 0.46^d^	11.89 ± 0.15^c^	23.99 ± 1.45^b^	13.67 ± 1.63^c^	28.28 ± 3.56^a^	2000 ^[D]^	<0.1	Bitter almond, nut ^[B]^
**Ketones**										
2-Octanone	1287	1.81 ± 0.26^d^	2.58 ± 0.02^d^	2.12 ± 0.30^d^	6.49 ± 0.00^c^	74.47 ± 1.58^a^	39.07 ± 1.69^b^	5 ^[N]^	>1	Green, fruity ^[N]^
6-Methylhept-5-en-2-One	1338	5.07 ± 1.04^a^	3.76±0.27bca	2.42 ± 0.42^d^	2.49±0.32dc	3.53±0.43cdb	3.83±0.01ba	0.068 ^[N]^	>1	nf
Acetoin	1284	0.53 ± 0.08^c^	2.58 ± 0.02^c^	320.72 ± 38.45^b^	551.65 ± 50.23^a^	48.36 ± 2.81^c^	58.36 ± 5.41^c^	150000^[I]^	<0.1	Buttery, fatty ^[I]^
Acetol	1308	ND	ND	70.65 ± 4.57^a^	16.01 ± 0.02^b^	ND	ND	nf		nf
**0thers**										
2,5-Dimethyl-hexane	1640	3.51 ± 0.44^d^	1.70 ± 0.01^d^	15.67 ± 2.09^c^	19.50±0.19cb	25.14±5.67ba	28.56 ± 2.13^a^	nf		nf
2,4-Di-tert-butylphenol	2318	2.14 ± 0.28^d^	2.45 ± 0.40^d^	14.38 ± 0.01^c^	93.45 ± 4.43^a^	14.12 ± 0.28^c^	22.84 ± 0.18^b^	200 ^[J]^	0.1–1	Phenolic^[A]^
Phenol	2000	0.56 ± 0.00^d^	0.80±0.18dc	1.22 ± 0.11^c^	1.98 ± 0.11^b^	2.98 ± 0.37^a^	0.82±0.07dc	31 ^[Q]^	<0.1	Phenolic ^[L]^

*Data are expressed as the means ± standard (*n* = 3). The different lowercase letters in each row indicate a significant difference between the samples (*P* < 0.05). ND, not detected. ^1^Odor threshold and odors were obtained from literatures: [A] (Tao and Li, 2009); [B] (Peng et al., 2013); [C] (Ferreira et al., 2000); [D] (Noguerol et al., 2009); [E] (Zea et al., 2001); [F] (Zhang et al., 2010); [G] (Niu et al., 2011); [H] (Mayr et al., 2014); [I] (Peinado et al., 2006); [J] (Gómez et al., 2007); [K] (Song Y. et al., 2017); [L] (López et al., 2004); [M] (Culleré et al., 2004); [N] (Zhao C. et al., 2020); [P] (Mihail et al., 2019); [O] (Guth, 1997); [P] (Du et al., 2011); [Q] (Gemert, 2011). ^2^OAV was calculated by dividing concentration by the odor threshold value of the compound. The scope of OAV is shown but not the specific value. The nf represented not found.*

The esters mainly included isoamyl acetate, ethyl lurate, ethyl decanoate, ethyl acetate, hexyl acetate, ethyl hexanoate, and ethyl octanoate. A large amount of acetate esters and ethyl esters were produced because a high concentration of alcohol and the related acetyl-CoA and acyltransferases interacted during spontaneous fermentation. Moreover, the produced wine contained high levels of ethanol, isoamyl alcohol, 1-hexanol, and phenylethyl alcohol. Our wine was also rich in hexanoic acid, octanoic acid, and decanoic acid. These esters, alcohols, and acids mainly conferred the overall flavor of our Petit Manseng wine. Aldehydes, ketones, and other volatiles may have also contributed to its flavors.

**FIGURE 2:**
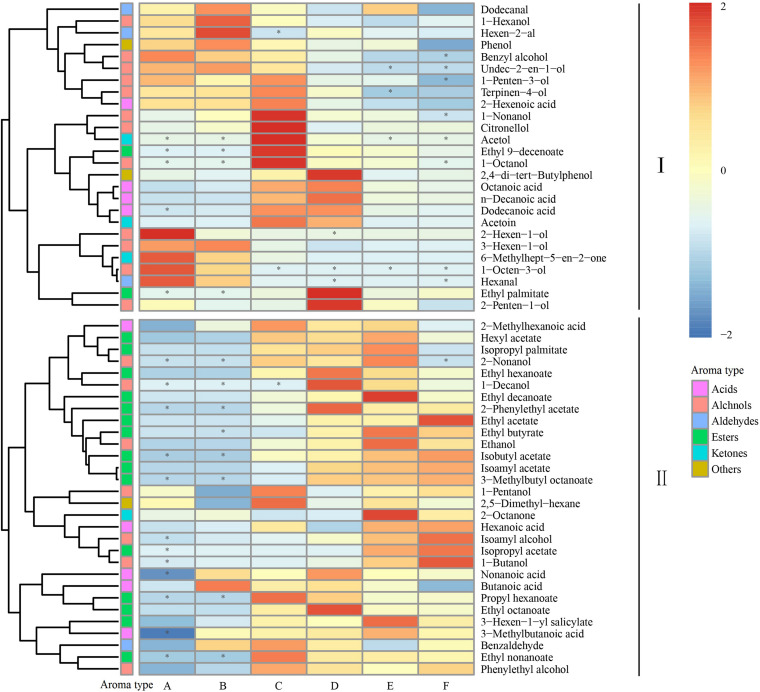
Heatmap cluster analysis of the volatile components during the fermentation. The asterisk indicates the substance with *P* < 0.05. Samples collected on fermentation days 0, 1, 4, 7, 11, and 14, respectively (for interpretation of the references to color in this figure legend, the reader is referred to the Web version of this article).

To better understand the dynamic changes in these volatile compounds, we analyzed the aroma components throughout the spontaneous fermentation process (Figure 2). The volatile compounds were divided into two classes based on the trends during the fermentation process: Class I contained 26 aromatic compounds (mainly including higher alcohols, C_6_ compounds, and fatty acids) and Class II contained 30 aromatic compounds (mainly including benzene derivatives, ethyl esters, acetic esters, and other volatiles). The high content of acetic and ethyl esters is responsible for the fruity flavor in the wine, whereas excessive concentrations of higher alcohols and fatty acids lead to unpleasant green, pungent, and rancid flavor in the wine. Based on our results, the spontaneous fermentation of Petit Manseng might produce wines with a more coordinated wine aroma (Shi et al., 2019).

**FIGURE 3:**
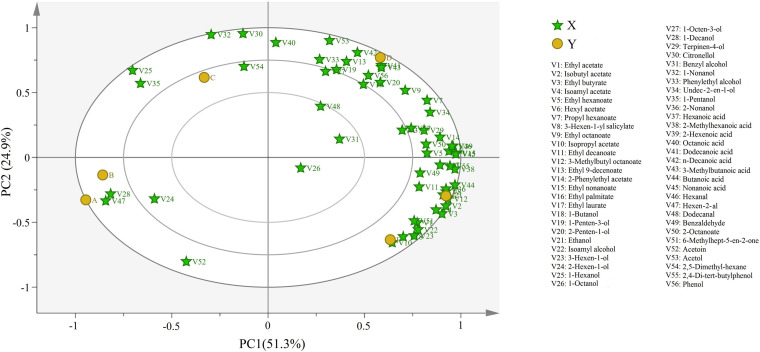
Bioplot of PCA for volatile compound of wine samples. A, B, C, D, E, and F represented samples collected on fermentation days 0, 1, 4, 7, 11, and 14, respectively.

We next used principal component analysis (PCA) to understand the correlation and segregation of aroma compounds in different wine samples (Figure 3). Here, 76.2% of the variance was explained by 56 different components, with PC1 and PC2 accounted for 51.3 and 24.9% of the variance, respectively. Most volatile compounds clustered at stages D and F, which corroborated the results indicating higher concentrations of volatile compounds in the wine. The rest of the wine samples clustered with only a few volatile compounds, also consistent with their lower concentration of volatile compounds in the respective stages. The variations in the location indicated that the aroma of Petit Manseng wine differed by the fermentation stage. Notably, half of the volatile compounds at stage D (i.e., alcohols and esters) were all located in the upper right quadrant.

### Sensory Analysis of Wine

**FIGURE 4:**
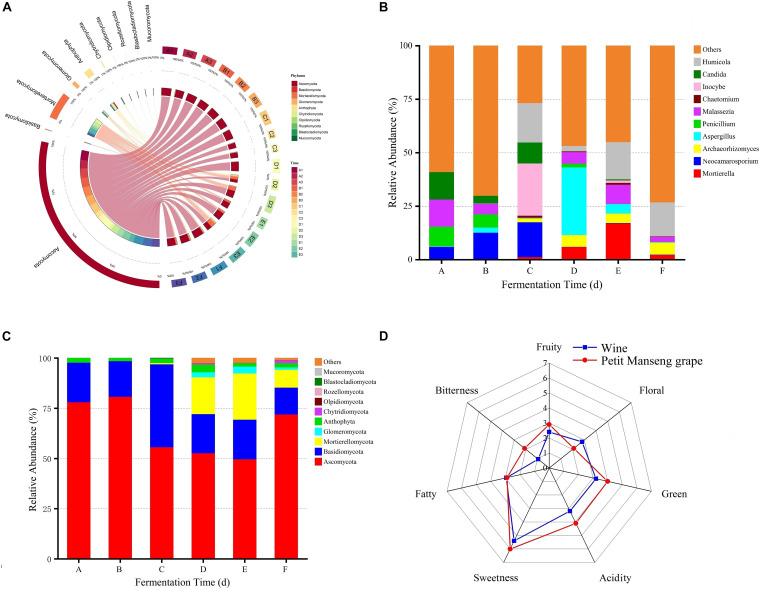
The relative abundance between fungal groups at the phylum **(A)** level, the relative abundance of the top 10 fungi at the genus **(B)** level, the relative abundance of top 10 fungi at the phylum **(C)** level, and sensory analysis of wine samples **(D)**. A, B, C, D, E, and F represented samples collected on fermentation days 0, 1, 4, 7,11, and 14, respectively.

The average scores of the sensory descriptions are presented in Figure 4D. Obvious differences were recorded in Petit Manseng grapes and wine fermented by Petit Manseng. The bitterness, green, acidity, and sweetness of the grapes scored higher than wine. The scores were almost similar of Petit Manseng grape and wine. Interestingly, the wine was noted to produce slightly stronger floral sensations than our grapes after spontaneous fermentation. These results indicated that our Petit Manseng wine has great market potential.

### Richness and Diversity Analysis

**FIGURE 5:**
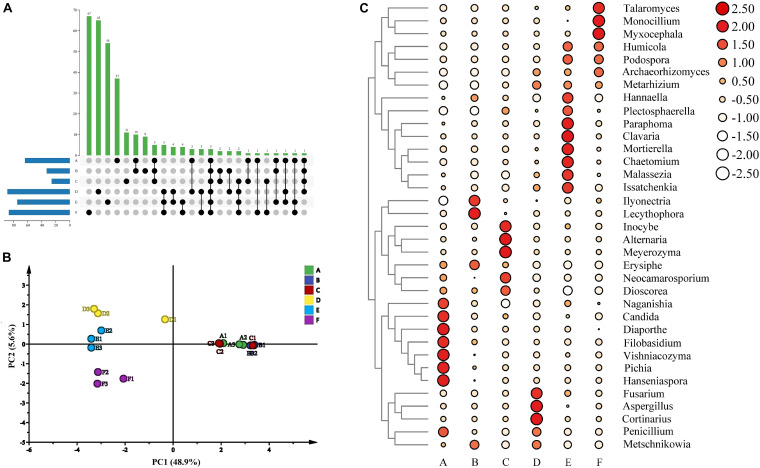
The upset diagram of the fungi genus among samples **(A)**, PCA of wine samples **(B)**, abundance clustering heatmap of major fungi genera in zymotic fluids collected from different fermentation times **(C)**. The scale bar shows the relative abundance (log scale) of each genus within a sample. A, B, C, D, E, and F represented samples collected on fermentation days 0, 1, 4, 7,11, and 14, respectively.

A total of 844,661 sequences for the fungal communities were clustered into 643 OTUs at a 97% identity threshold. The rarefaction curves of all samples tended to be flat, indicating that the amount of our sequencing data was reasonable (Supplementary Figure 1). For fungal communities, the richness in stage F was the highest according to the Chao1 and ACE indexes; stage F also showed the highest diversity based on the Shannon and Simpson indexes (Supplementary Table 1). Moreover, the values of the Chao1, ACE, Shannon, and Simpson indexes at stages E and F were higher than those at other stages, whereas Robbins index was the highest at stage C among all the stages (Supplementary Figure 2). Therefore, the fungal richness and diversity of the wine samples from different fermentation stages showed differences according to the alpha indexes. In addition, this study described the common and unique OTUs of the microorganisms from different fermentation stages. There were 37, 9, 11, 65, 54, and 67 fungal OTUs noted in stages A, B, C, D, E, and F, respectively, and 3 OTUs were shared among all six fermentation stages (Figure 5A). Therefore, the structure of the fungal communities in the sweet wine of Petit Manseng demonstrated differences based on the fermentation stage. Moreover, we investigated the dynamic changes in fungal communities during different fermentation periods. Although the original OTU data showed that the total number of OTUs increased as fermentation continued, the number of cultivable fungi gradually decreased (Supplementary Table 2). The changes corresponding to the fermentation stages, along with changes in environmental factors, led to changes in fungal adaptability. Four colony micrographs (Supplementary Figure 4), five genera, and seven species of yeast were isolated in the spontaneous fermentation process based on the phylogenetic tree (Supplementary Figure 7).

Our beta diversity analysis of the fungal structure based on PCA during wine fermentation indicated that the contribution rate of PC1 was 48.9%, whereas that of PC2 was 5.6% (Figure 5B). We found that the structure of fungal composition was clustered together at stages A, B, and C and was very large at stages D, E, and F. These results contradicted the previous results, possibly because the previous study performed incomplete fermentation of Petit Manseng wine (Chen et al., 2020). We also noted some unusual dissimilarities. For example, our D1 deviated from the main clusters. In summary, our data indicated that fungal community abundance was dependent on the fermentation stage.

### Microbial Abundance During Spontaneous Fermentation

By comparing the fungal communities in wine samples from different fermentation stages, we found that the main phyla appeared in all samples, but their content varied (Figure 4A). In particular, the top 10 relatively abundant fungal phyla included Ascomycota, Basidiomycota, Mortierellomycota, Glomeromycota, Anthophyta, Chytridiomycota, Olpidiomycota, Rozellomycota, Blastocladiomycota, and Mucoromycota (Figure 4C). The distinction of fungal abundance was obvious in different fermentation stages. For example, the relative abundance of Ascomycota was 78, 81, 56, 53, 50, and 72% in stages A, B, C, D, E, and F, respectively. Moreover, fungal abundance was confirmed to be affected by the fermentation stage.

The extracted high abundance of fungal OTUs as well as the distribution of six different phylum-to-species species is illustrated in Supplementary Figure 3. The result showed that Ascomycota had a denser species distribution than other phyla, whereas Glomeromycota showed the opposite trend. At the fungal genus level, *Candida* and *Malassezia* had the highest percent relative abundance at stage A. These results differed from those reported previously, possibly because of differences in the grape varieties and origins used (Sirén et al., 2019). Moreover, *Candida* and *Malassezia*, the abundant genera, dominated in the early and middle stages of fermentation (Figure 4B), whereas *Mortierella* and *Humicola* dominated the later fermentation stages. The relative abundance of *Aspergillus* demonstrated notable fluctuations, which may be the reason for the increased alcohol concentration. The relative abundance of *Penicillium* peaked at stage A and then decreased rapidly. Moreover, *Vishniacozyma*, *Hanseniaspora*, and *Pichia*, as dominant fungi present on the grapes in China’s vine-growing regions, were found in our wine during spontaneous fermentation (Li et al., 2010). Regarding the fungal communities, *Hanseniaspora*, *Pichia*, *Vishniacozyma*, *Filobasidium*, *Diaporthe*, and *Candida* were the dominant taxa at stage A (Figure 5C). In addition, *Lecythophora*, *Inocybe*, *Alternaria*, *Meyerozyma*, and *Erysiphe* dominated stages B and C, whereas at stages E and F, the dominant taxa were *Humicola*, *Podospora*, *Archaeorhizomyces*, and *Metarhizium*. Furthermore, *Metschnikowia* and *Issatchenkia* were detected in the fermentation process, with a strong positive correlation at stages D and E. *Metschnikowia* and *Issatchenkia* may also be responsible for the increased alcohol contents (Liu et al., 2016).

### Co-occurrence and Exclusion Analysis Reveals the Relationships Among Different Microorganisms

**FIGURE 6:**
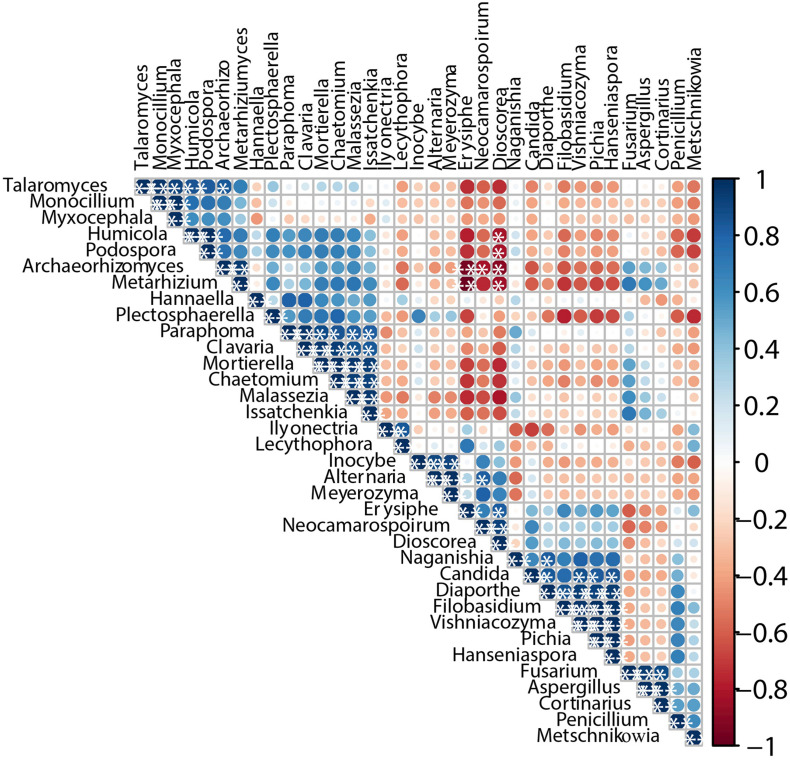
Co-occurrence and co-exclusion relationship analysis of fungi. The figure presents a Pearson’s rank correlation matrix of the top 35 fungi genera abundance. Strong correlations are indicated by large circles, whereas weak correlations are indicated by small circles. The color of the scale bar denotes the nature of the correlation, with 1 indicating a perfect positive correlation (dark blue) and -1 indicating a perfect negative correlation (dark red). Only significant correlations (| r| > 0.7, FDR < 0.05) are shown with *.

The interaction of microorganisms is considered an important factor supporting the structure of microbial communities (Huang et al., 2018). We calculated Pearson’s rank correction coefficients to represent beneficial or antagonistic relationships between the dominant microorganisms. The correlations of different fungi are displayed in Figure 6. *Issatchenkia* showed weak exclusion from other fungal genera except for *Hannaella*, *Naganishia*, *Fusarium*, *Aspergillus*, and *Cortinarius* (*P* < 0.05). In addition, *Dioscorea* and *Erysiphe* showed obvious exclusion toward *Talaromyces*, *Humicola*, *Podospora*, *Archaeorhizomyces*, and *Metarhizium*. In contrast, *Paraphoma*, *Clavaria*, *Mortierella*, and *Chaetomium* demonstrated strong co-occurrence with *Malassezia* and *Issatchenkia*, and *Pichia* and *Hanseniaspora* presented strong co-occurrence with *Diaporthe*, *Filobasidium*, and *Vishniacozyma* (*P* < 0.05). Thus, most genera present during spontaneous fermentation presented weak co-occurrence patterns.

### Correlation Analysis Between Core Microbiota and Volatile Compounds

**FIGURE 7:**
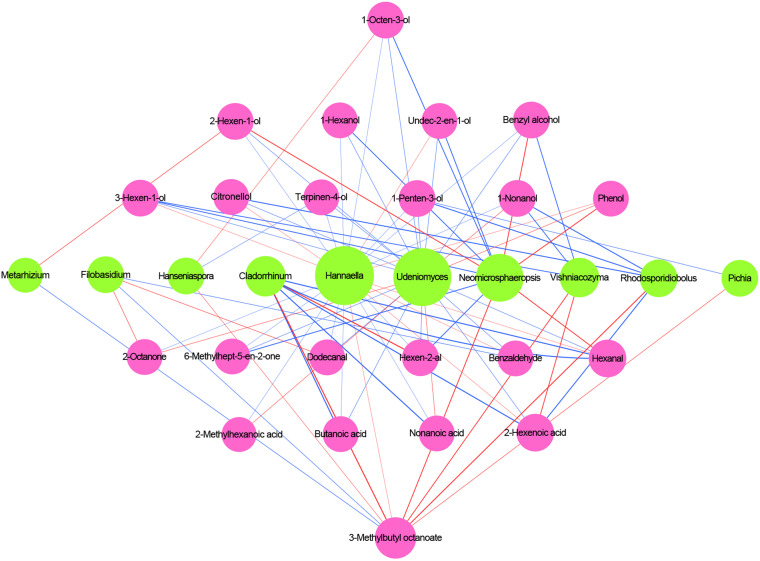
Correlation analysis of microbiota and volatile compounds during spontaneous fermentation. Core fungal genera are shown in green and core volatile compounds are shown in purple. Blue lines indicate positive correlations, and red lines indicate negative correlations.

The correlation of core microbiota and volatile compounds were analyzed during the Petit Manseng wine fermentation process, as shown in Figure 7. Three criteria were used to discover the relationship between the microbiota and the aroma compounds: (1) a relatively stable abundance was maintained throughout the fermentation process, (2) the variable importance for the predictive component (VIP) values of microorganisms and volatiles were >1.0, and (3) the absolute values of linear correlation coefficient (R) between the concentration of volatile compounds and the relative abundance of microbial were >0.7 (He et al., 2020). The changes in VIP values for the fungal group at the genus level and the volatile compounds are shown in Supplementary Figures 5, 6, respectively.

According to the aforementioned criteria, 10 microbial genera were regarded as key microorganisms in spontaneous fermentation. *Udeniomyces*, *Hannaella*, and *Neomicrosphaeropsis* presented high correlations with alcohol and acids, such as 1-octen-3-ol and nonanoic acid. *Vishniacozyma* correlated with five core volatile compounds, of which it was positively correlated with 3-hexen-1-ol, 1-nonanol, and benzyl alcohol. *Cladosporium* negatively correlated with hexen-2-al and 3-methylbutyl octanoate. Non-*Saccharomyces cerevisiae* yeasts commonly found in wine, such as *Hanseniaspora* and *Pichia*, were positively correlated with some alcohols. *Metarhizium* presented characteristics similar to *Filobasidium* concerning 3-methylbutyl octanoate. In general, most fungal genera, except *Metarhizium* and *Filobasidium*, were negatively correlated with 3-methylbutyl octanoate. Whether the aforementioned microorganisms can produce and absorb related volatile compounds warrants further study.

Overall, the changes in the microbial community differed throughout the spontaneous fermentation process of Petit Manseng. As reported previously, wine quality is related to the metabolic activity and fermentation behaviors of the yeasts (Antonelli et al., 1999). Compared with *S. cerevisiae*, non-*Saccharomyces* yeasts have gained more attention because they produce esterase, β-glucosidase, lipase, and protease and thus increase the amount of active aroma substance (Escribano et al., 2017).

In this study, *Hanseniaspora*, *Vishniacozyma*, and *Pichia* were significantly correlated with at least one volatile compound during spontaneous fermentation. Based on our multivariate analysis results, the correlation between microorganisms and volatile compounds was evident. This requires further research using multiomics methods to verify the association between core microorganisms and specific flavors (Huang et al., 2018).

## Discussion

Our Petit Manseng wine was produced *via* spontaneous fermentation and had a final alcohol concentration of 12.01% (v/v), accompanied by a quick decrease in the total sugar content from 345.35 to 110.84 g/L. The gradually accumulated ethanol inhibited yeast metabolism and the ability to produce ethanol.

With regard to organic acids, *Pichia* can metabolize lactic acid to pyruvate and then to acetyl-CoA and acetaldehyde during liquor production (Song Z. et al., 2017). The microorganisms that accumulated citric acid mainly include some fungi from the genera *Aspergillus*, *Candida*, and *Penicillium* (Papagianni, 2007). Tartaric acid is resistant to degradation and metabolism by wine microorganisms (Torija et al., 2003). In this study, the wine tartaric acid concentration decreased slowly because it was precipitated as potassium and calcium salts during fermentation.

In the present study, we identified 56 volatile compounds linked to spontaneous wine fermentation. Esters, which can increase the complexity of volatiles with the extension of the fermentation stage, are indispensable components of wine (Guo et al., 2020). The concentrations of fatty acid ethyl esters, ethyl butyrate, ethyl hexanoate, ethyl octanoate, ethyl decanoate, and ethyl laurate initially increased and then showed a downward trend. In contrast, the contents of the acetic esters like isoamyl acetate, isopropyl acetate, and 2-phenylethyl acetate increased during the fermentation process. The odor activity values (OAVs) of these compounds were >1 at the end of spontaneous fermentation; this observation may be related to high acyltransferase and alcohol acetyltransferase expression during spontaneous fermentation (Saerens et al., 2006; Procopio et al., 2011).

Alcohols represented one of the largest groups of volatile compounds noted during spontaneous fermentation, and they included 1-hexanol, phenylethyl alcohol, isoamyl alcohol, and ethanol. The contents of all alcohols except ethanol gradually increased during the fermentation process. However, these alcohols (1-hexanol and phenylethyl alcohol) do not positively enhance the wine flavor wines because of their relatively high odor threshold. In contrast, ethanol with a low threshold showed an OAV of >1. Moreover, citronellol’s OAVs were also >1 and influenced the fruity character of the wine (Pérez et al., 2018). Acids, considered important for wine fermentation, were produced *via* yeast metabolism during spontaneous fermentation. These acids primarily comprised hexanoic acid and octanoic acid and flocked in the late stage of spontaneous fermentation, with a total content of 3448.90 μg/L.

Aldehydes, the significant source of herbaceous in wine, mainly came from fatty acid oxidation and amino acid degradation due to microbial fermentation (Zhao G. et al., 2020). Benzaldehyde was a widely used aromatic aldehyde, with an OAV of <0.1 at the end of fermentation. Fatty acid release and flavor substance catabolism *via* β-oxidation led to ketone formation (Collins et al., 2003). 2-Octanone actively contributed to the fruity of wine, with an OAV of >1.

The OAVs of the trace compounds formed during spontaneous fermentation were <1. In addition, most of these volatile compounds showed a concentration increase throughout wine fermentation. Although these compounds were present at a relatively low concentration, some of them had an indirect effect on the flavor and countered the organoleptic properties of the wine (Asproudi et al., 2018).

Changes were also noted in the indigenous microorganisms during the spontaneous fermentation process. The microbial communities participating in initial spontaneous alcoholic fermentation are inherently present in Petit Manseng grapes. Nevertheless, many other microorganisms also participate in this process. Therefore, the relative abundance of fungal communities during spontaneous fermentation demonstrated obvious fluctuations, possibly also reflecting the effects of the ethanol fermentation environment. Wang and Liu (2013) reported fungal communities were inhibited by harsh conditions until they became tolerant or adapted to the harsh fermentation environments. In the current study, the abundance of *Candida* reduced during spontaneous fermentation due to its sensitivity to ethanol, similar to that described in previous studies (Heard and Fleet, 1985). Notably, *Humicola* began to appear at stage C and gradually increased in abundance at the end fermentation. This result was expected because *Humicola* was resistant to ethanol (Bassey et al., 2017). Some *Humicola* species positively contributed flavor by producing various enzymes, thus indicating many of their potential applications in various industries (Bokhari et al., 2010). *Aspergillus* was present throughout the fermentation process with different relative abundance values. Their abundance peaked in the middle stage of the fermentation. *Aspergillus* has powerful environmental adaptability, and it is resistant to acids and ethanol (Freire et al., 2017; Sheng et al., 2018). Some fungi such as *Pichia*, the important producers of various secondary metabolites, occurred in the early spontaneous fermentation period of our wine. This result indicated a potential application of these microorganisms in producing many aroma substances to improve the quality of Petit Manseng wine.

The relationship between volatile compounds and microbial succession dynamics during the spontaneous fermentation of Petit Manseng wine remains unclear. We thus investigated the key functional microorganism responsible for the generation of aroma compounds. We found that *Hannaella*, *Udeniomyces*, and *Neomicrosphaeropsis* were the highest contributors to the generation of volatile compounds, especially during alcohol production. In addition, *Filobasidium* and *Hannaella* abundance was negatively correlated with 2-octanone production. Moreover, *Vishniacozyma* produced antibiotic compounds or enzymes to maintain their niches (Gramisci et al., 2018). This was related to the formation of aldehydes, such as hexanal, and acids, such as 2-hexanoic acid, during fermentation.

In this study, the indigenous microbial of wine grapes reflect the health of grapes and play an indispensable role in wine flavor and quality. To the best of our knowledge, this is the first study of microbial succession during the spontaneous fermentation of sweet wine fermented by Petit Manseng using HTS technology. Additional studies on the indigenous non-*Saccharomyces* species detected in this study may aid in understanding their role during spontaneous fermentation, their contribution to the sensory quality of sweet wine, and microbial safety.

## Conclusion

Spontaneous fermentation of Petit Manseng could strengthen its aromatic profile. Fungi, as a significant part of wine, were responsible for the formation of the characteristic aroma and volatile compounds (including many esters and alcohols) in the wine. The results of this study may be used as a reference for producing Petit Manseng sweet wine with typical characteristics.

## Data Availability Statement

The data presented in the study are deposited in the National Center for Biotechnology Information (NCBI) repository, accession number PRJNA748614 (https://www.ncbi.nlm.nih.gov/bioproject/PRJNA748614). The accession numbers for dataset of our 18 samples that have been generated or analyzed in the study are: SRX11512770, SRX11513000, SRX11513001, SRX11513002, SRX11513003, SRX11513004, SRX11513005, SRX11513006, SRX11513007, SRX11513008, SRX11513-009, SRX11513010, SRX11513011, SRX11513012, SRX11513013, SRX11513014, SRX11513015, and SRX11513016.

## Author Contributions

YM wrote the main text of the manuscript and performed the experiment. BW analyzed the fungi data. TL, XX, YJ, and XJ analyzed the volatile component data. XS supervised the research activities. All authors reviewed the manuscript.

## Conflict of Interest

The authors declare that the research was conducted in the absence of any commercial or financial relationships that could be construed as a potential conflict of interest.

## Publisher’s Note

All claims expressed in this article are solely those of the authors and do not necessarily represent those of their affiliated organizations, or those of the publisher, the editors and the reviewers. Any product that may be evaluated in this article, or claim that may be made by its manufacturer, is not guaranteed or endorsed by the publisher.
