# Supplementary material for: Investigation of Volatile Compounds, Microbial Succession, and Their Relation During Spontaneous Fermentation of Petit Manseng
Source: Front Microbiol. 2021 Aug 12;12:717387. doi: 10.3389/fmicb.2021.717387 (PMC8406806; doi:10.3389/fmicb.2021.717387)
Supplement: Supplementary file 1 [file Data_Sheet_1.PDF]

Supplementary materials for:

Investigation of volatile compounds, microbial succession and their relation during spontaneous fermentation of Petit Manseng

Yanqin Ma, Tian Li, Xiaoyu Xu, Yanyu Ji, Xia Jiang, Xuewei Shi*, Bin Wang*

Food college, Shihezi University, Shihezi 832000, Xinjiang Uygur Autonomous Region, P. R. China.

* Corresponding authors

E-mail addresses: B. W. : binwang0228@shzu.edu.cn; X. S. : shixuewei@shzu.edu.cn

Tel.: 86-0993-2058093

Catalog

Supplementary tables.. 1
Table S1. Richness and diversity indexes of fungi communities in the wine............................ 1
Table S2. Distribution of fungi in different fermentation periods..2
Supplementary figures..3
Figure S1. Rarefaction curve of assessing sequencing saturation..3
Figure S2. Alpha diversity indexes (a: Chao 1, b: ACE, c: Shannon, d: Simpson) of fungi diversity... 3
Figure S3. The dynamic change of fungi relative abundance: the maptree of phylum to species.. 4
Figure S4. Colony micrographs of yeast colony...4
Figure S5. VIP (variable importance for predictive components) plot of fungal community....5
Figure S6. VIP (variable importance for predictive components) plot of volatile compounds. 5
Figure S7. Phylogenetic tree of fungal ITS sequence based on Neighbor-Joining method.......6

Supplementary tables

Table S1. Richness and diversity indexes of fungi communities in the wine.

Sample	Richness	Chao1	ACE	Shannon	Simpson	Robbins
A	98.33±47.51^{a}	112.78±47.21^{a}	112.26±49.25^{a}	2.33±0.11^{b}	0.77±0.01^{b}	0.16±0.02^{b}
B	114.67±24.91^{b}	152.82±7.44^{a}	139.95±13.25^{a}	1.56±0.06^{c}	0.60±0.01^{c}	0.22±0.06^{c}
C	141.00±54.01^{a}	170.41±66.47^{a}	170.17±62.12^{a}	1.67±0.30^{b}	0.65±0.05^{b}	0.24±0.04^{b}
D	153.67±91.19^{a}	174.34±92.84^{a}	176.00±95.44^{a}	3.11±0.84^{b}	0.91±0.09^{b}	0.20±0.05^{b}
E	172.67±30.35^{a}	194.73±25.59^{a}	197.04±16.53^{a}	3.19±0.22^{b}	0.94±0.03^{b}	0.20±0.09^{b}
F	208.67±33.08^{b}	239.96±23.47^{ab}	247.89±20.35^{a}	3.31±0.42^{c}	0.94±0.01^{c}	0.23±0.09^{c}

Data are expressed as the means ± standard (n=3). The different lowercase letters in each row indicate a significant difference between the samples ($P<0.05$).

Table S2. Distribution of fungi in different fermentation periods

Colony morphology		Strain number
YPD medium	WL medium	
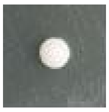	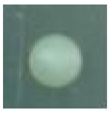	A9, A2, B12, B3, C9, C5, C6, D1, E4, F4, F5
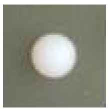	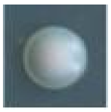	A4, B4, A14, B11, B3, C4, D4, E2, E3, E5, F1
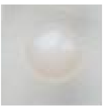	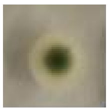	A3, B4, B1, E5
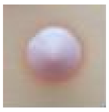	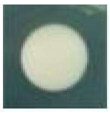	A10, A8, A15, B9, C1, C3
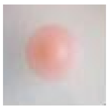	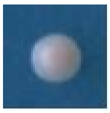	A5, A7, B5, B6,C7, D5
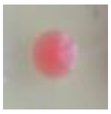	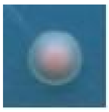	A6, A12, A13, B10,C10
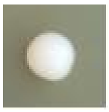	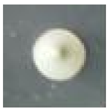	A1, A11, B7, B8,D2, D3, E1, F2, F3

A, B, C, D, E, F represented 0d, 1d, 4d, 7d, 11d, 14d.

Supplementary figures

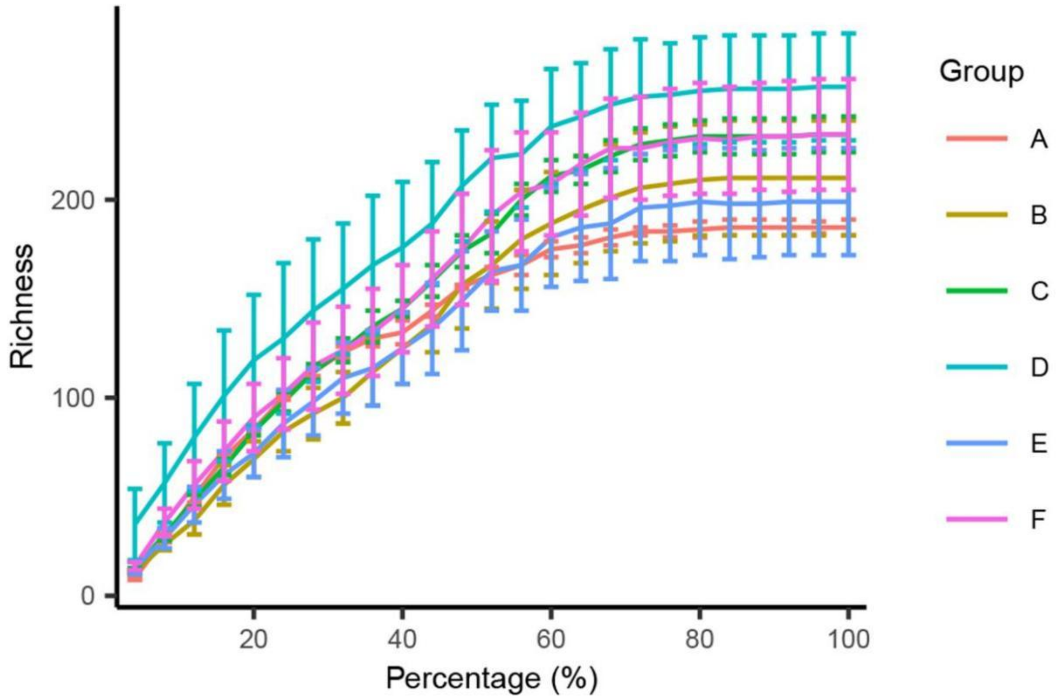

Figure S1. Rarefaction curve of assessing sequencing saturation.

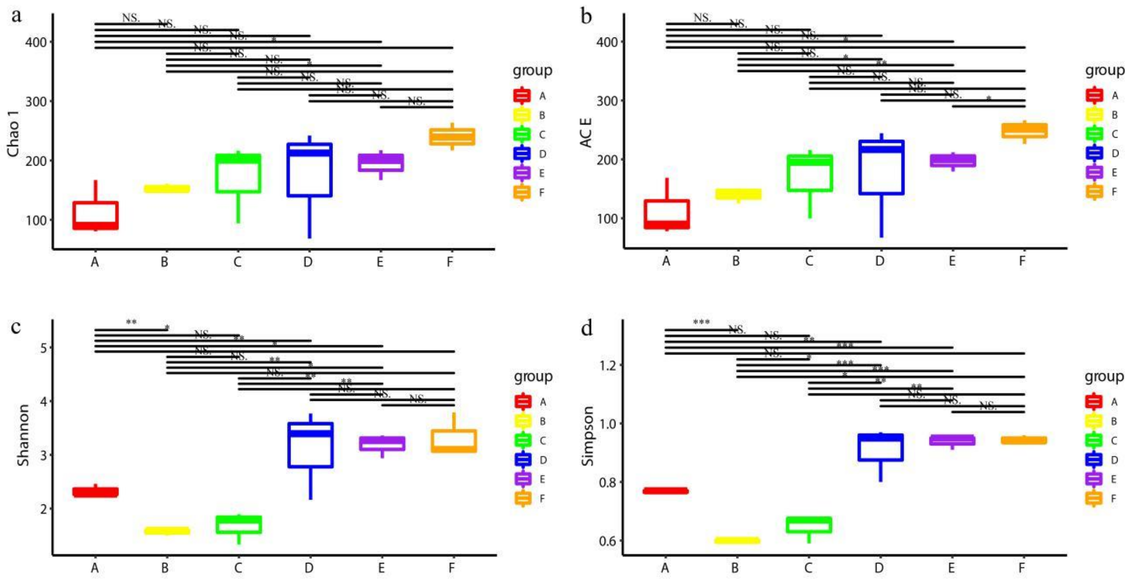

Figure S2. Alpha diversity indexes (a: Chao 1, b: ACE, c: Shannon, d: Simpson) of fungi diversity.

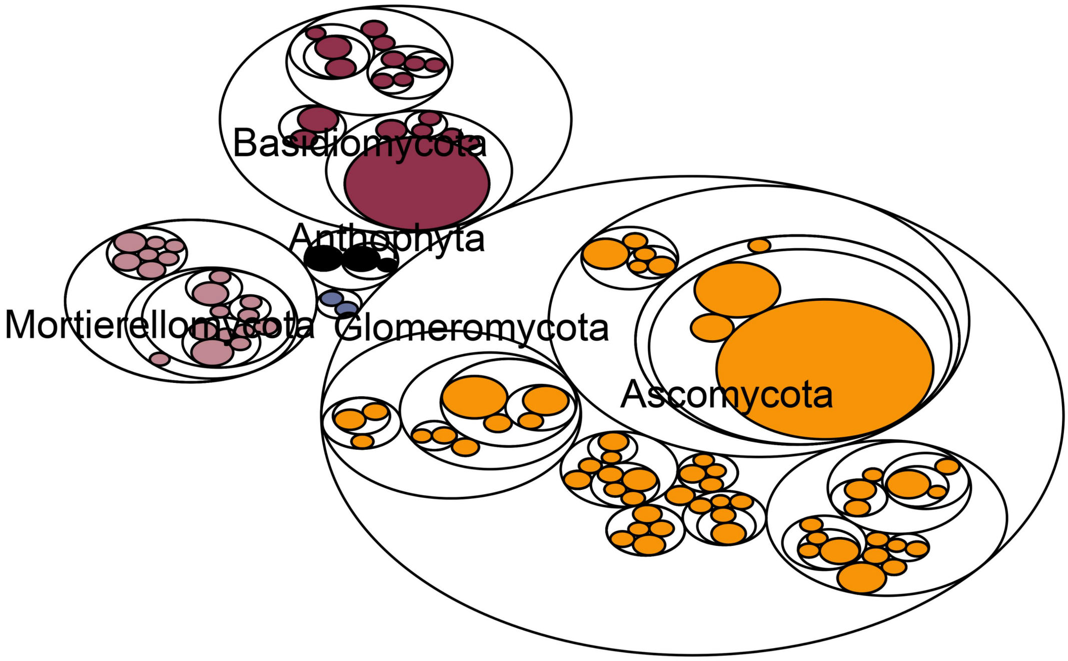

Figure S3. The dynamic change of fungi relative abundance: the maptree of phylum to species.

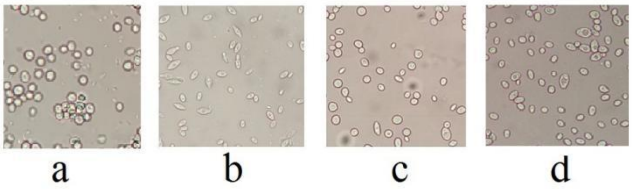

Figure S4. Colony micrographs of yeast colony.

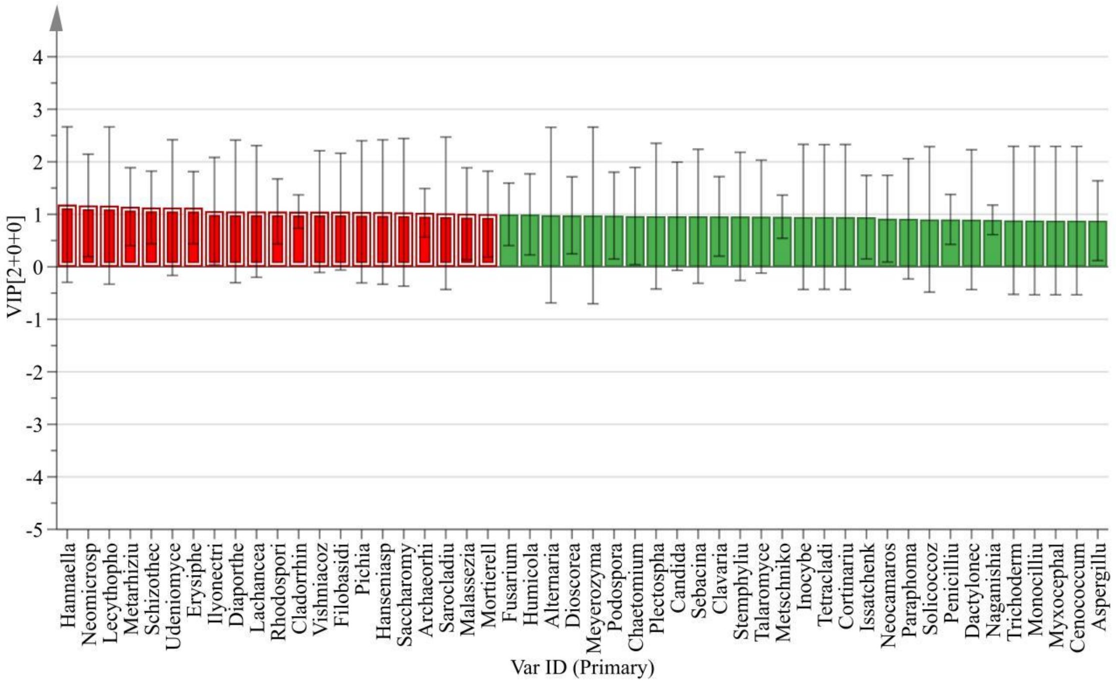

Figure S5. VIP (variable importance for predictive components) plot of fungal community.

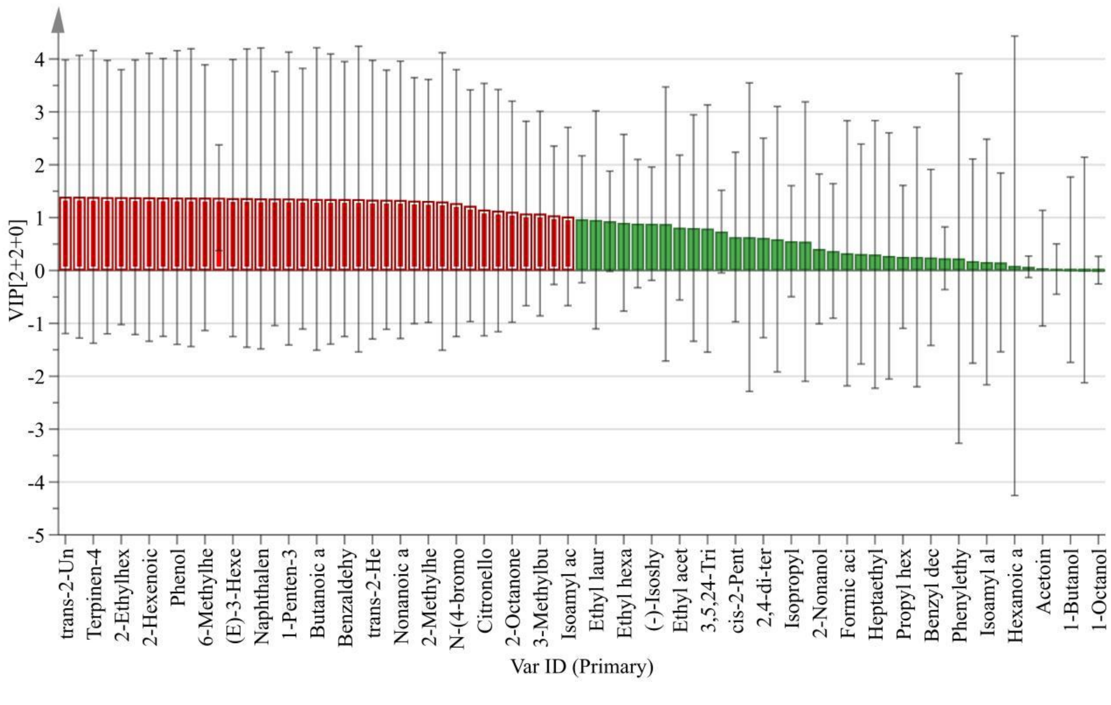

Figure S6. VIP (variable importance for predictive components) plot of volatile compounds.

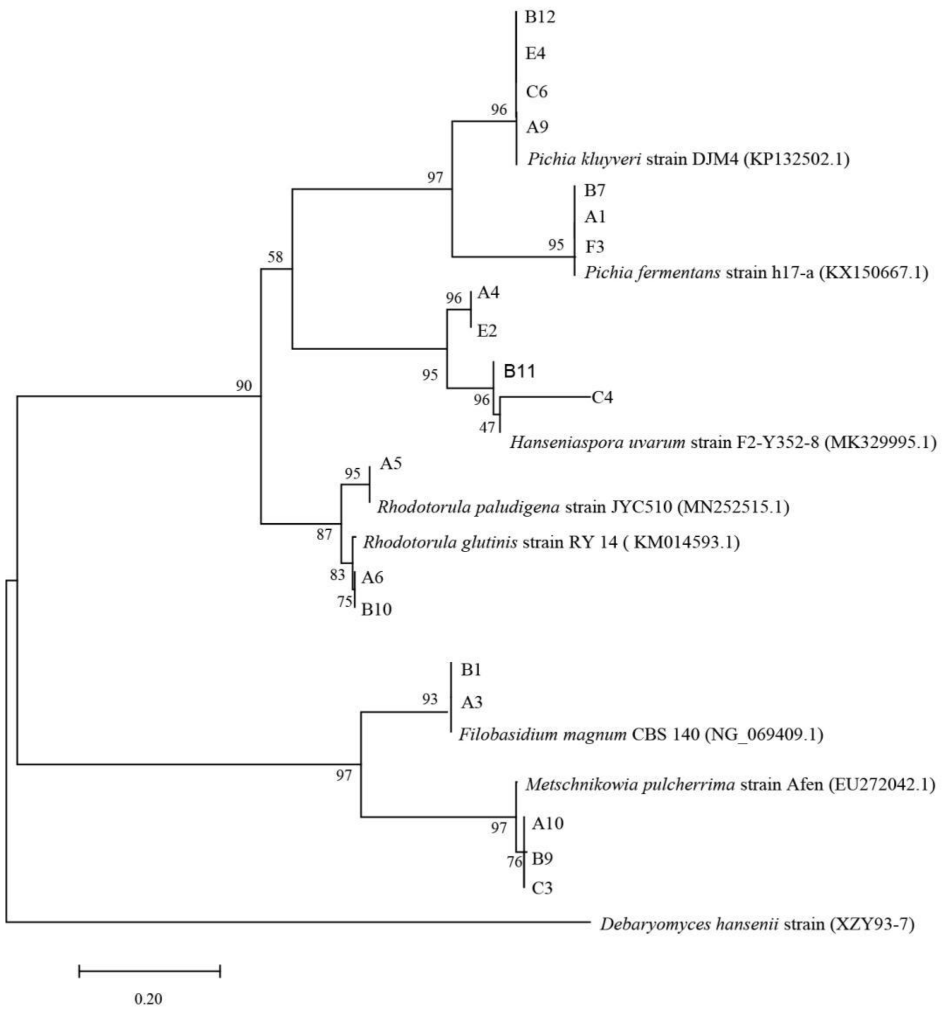

Figure S7. Phylogenetic tree of fungal ITS sequence based on Neighbor-Joining method.
